# Types, Causes, Detection and Repair of DNA Fragmentation in Animal and Human Sperm Cells

**DOI:** 10.3390/ijms131114026

**Published:** 2012-10-31

**Authors:** Clara González-Marín, Jaime Gosálvez, Rosa Roy

**Affiliations:** 1 Sexing Technologies, 22575 State Highway 6 South, Navasota, TX 77868, USA; E-Mail: cgonzalez@sexingtechnologies.com; 2 Biology Department, Universidad Autonoma of Madrid, C/ Darwin nº 2. 28049 Madrid, Spain; E-Mail: jaime.gosalvez@uam.es

**Keywords:** sperm, infertility, DNA fragmentation, DNA repair, oocyte

## Abstract

Concentration, motility and morphology are parameters commonly used to determine the fertilization potential of an ejaculate. These parameters give a general view on the quality of sperm but do not provide information about one of the most important components of the reproductive outcome: DNA. Either single or double DNA strand breaks can set the difference between fertile and infertile males. Sperm DNA fragmentation can be caused by intrinsic factors like abortive apoptosis, deficiencies in recombination, protamine imbalances or oxidative stress. Damage can also occur due to extrinsic factors such as storage temperatures, extenders, handling conditions, time after ejaculation, infections and reaction to medicines or post-testicular oxidative stress, among others. Two singular characteristics differentiate sperm from somatic cells: Protamination and absence of DNA repair. DNA repair in sperm is terminated as transcription and translation stops post-spermiogenesis, so these cells have no mechanism to repair the damage occurred during their transit through the epididymis and post-ejaculation. Oocytes and early embryos have been shown to repair sperm DNA damage, so the effect of sperm DNA fragmentation depends on the combined effects of sperm chromatin damage and the capacity of the oocyte to repair it. In this contribution we review some of these issues.

## 1. Introduction

Male infertility has traditionally been diagnosed by microscopic assessment of concentration, motility and morphology of sperm in the ejaculate. These tests are essential to provide the fundamental information on sperm quality. However, evidence based medicine shows that sperm DNA fragmentation (SDF) tests can also differentiate fertile from infertile males and that high levels of SDF are positively correlated with lower fertilization rates in IVF, impaired implantation rates and an increased incidence of abortion. Decades ago, Evenson *et al.*[1] suggested that the assessment of DNA integrity in sperm could be an independent marker of fertility. Clinical data followed, demonstrating higher levels of chromatin damage in men with severe sperm defects [2]. The negative impact of high levels of sperm DNA damage on both natural [3,4] and ART [5] have also been demonstrated. Subsequently, the assessment of DNA damage in the male germ line and the impact on reproductive outcome has received significant attention. In animals, where DNA damage can be experimentally induced in the paternal germ line, strong associations have been shown between damage to the paternal genome and embryo development including effects on the new born and subsequent generations [6,7]. These experiments are not feasible in humans, but provide clear warnings of the impact that some therapies like cancer treatments may exert on the sperm DNA.

Sperm chromatin differs from somatic cells in both constituents and arrangement. During spermiogenesis, protamines, which are half the size of histones, replace the majority of histones and the chromatin is wound into unique supercoiled structure named toroids [8]. As the sperm pass through the epididymis, the protamines are cross-linked by disulphide bonds reducing the chromatin to one-sixth the volume taken up in somatic cell nuclei. This dense compaction gives protection against exogenous assault to the sperm DNA [9]. Despite this protection, basal levels of sperm DNA damage are relatively high in infertile and even fertile men when compared with other species [10]. In addition to exhibiting higher basal levels of DNA damage, sperm from infertile males are more susceptible to damage over time after ejaculation [11].

Damaged DNA has been observed in testicular, epididymal and ejaculated sperm. Sperm DNA first becomes susceptible to damage if chromatin packing is not completed during spermatogenesis when protamine replacement is occurring in elongating spermatids. Temporary nicks, linked to the topoisomerases activity, facilitate histone-protamine replacement [12], but if these nicks are not fixed they will evolve into DNA fragmentation on mature sperm. Other internal causes of DNA fragmentation are abortive apoptosis, deficiencies in recombination or oxidative stress, among others. Damage can also occur due to extrinsic factors, the so called iatrogenic damage, and can be a result of storage temperatures, extenders used, handling conditions, lapse of time after ejaculation, infections, reaction to medicines, or post-testicular oxidative stress.

A number of tests are currently available to evaluate SDF. These include the Sperm Chromatin Structure Assay (SCSA) [3,13], the TUNEL Assay, the In Situ Nick Translation (ISNT) [14], the DNA Breakage Detection-Fluorescence in Situ Hybridization (DBD-FISH) test [15], the Comet Assay [16], the Single-cell pulsed-field gel electrophoresis technique [17] and the Sperm Chromatin Dispersion Test (SCDt) [18,19]. All of these are valuable tools to gain information on the state of the desoxirribonucleic acid in the sperm, but the existing mechanisms of reparation existing in the oocyte, may provide a confusing view of the real impact of the DNA damage on pregnancy. This is probably one of the main reason supporting discrepancies existing among authors about the impact of SDF on pregnancy rates. Some authors report SDF to be an important predictor of male infertility, especially that associated with compromised syngamy and early embryonic loss [20–22], although in other cases, this relationship is still not clear [23–26].

DNA repair does occur in developing sperm but it is terminated as transcription and translation stops post-spermiogenesis. As a result, sperm have no mechanism to repair DNA damage that occurs during their transit and storage in the epididymis or post-ejaculation. However, that ocytes and early embryos have been shown to repair some types of sperm DNA breakage. Consequently, the biological effect of abnormal sperm chromatin structure depends on the combined effects of level and type of sperm chromatin damage and the capacity of the oocyte to repair it.

This review will focus on sperm DNA single and double strand breaks caused by intrinsic and extrinsic factors. Additionally, some attention will be given to DNA damage response (DDR) mechanisms in spermatids and in the oocyte after fertilization.

## 2. Detection of DNA Damage: Techniques

As mentioned in the introduction, various tests are currently available to evaluate sperm DNA damage. An important aspect to assess DNA Fragmentation is related to the type of damage affecting the DNA strands and the susceptibility of that DNA to get fragmented. Tests like SCSA, SCDt or the Comet Assay at alkaline or acidic pH, require a denaturalization step to detect the DNA fragments or the potential breaks in the DNA. However, TUNEL, ISNT or Comet Assay at neutral pH do not require denaturalization and they measure real DNA breakage, either on one or both strands of the DNA. The important questions about all these methods are whether they reveal the same type of damage, whether they obtain comparable results and last but not least, whether they are standardized. The different data in the literature for the levels of SDF in fertile and subfertile men, and the lack of agreement among the different studies evaluating the impact of SDF on ART outcomes, reflect how different methods may affect the results. A systemic meta-analysis of papers reporting the relationship between sperm DNA damage and ART outcomes published by Li *et al*. [27] shows how, when data are pooled according to the method (TUNEL and SCSA) employed in the study, completely different conclusions can be drawn.

Two of the most employed techniques to reveal SDF are TUNEL and SCSA. Although the two techniques show correlated results, they are not equivalent and reveal different types of damage [28]. In particular, the TUNEL assay quantifies the amount of cellular DNA breakage by incorporating fluorescent dNTPs at single- and double-stranded DNA ends in the presence of the enzyme terminal deoxynucleotidyl transferase while the SCSA method determines the extent of cellular DNA denaturation (induced by acids or heat treatment) by measuring the metachromatic shift of acridine orange from green (indicative of intercalation into double-stranded DNA) to red fluorescence (indicative of association with single-stranded DNA).

Previous studies indicate that DNA fragmentation measured by SCSA is not related to fertilization rates, embryo quality, and pregnancy rates in *in vitro* fertilization and intra cytoplasmic sperm injection, but could be related to spontaneous abortion rates [29–31]. On the other hand, Studies by Greco *et al*. [32], showed that the microinjection of sperm with DNA fragmentation over 15% analyzed with TUNEL, resulted in a pregnancy rate of 5.6% *versus* a 44.4% when DNA fragmentation was below 6%.

Other methods frequently used in clinical investigations are the COMET (a single-cell gel electrophoresis assay) and the SCDt, which are relatively simple methods for detecting DNA damage in individual cells. Both methods consist of several steps: cells are embedded in agarose; lysis is carried out; DNA is stained and sperm are analyzed under the microscope. The COMET assay includes an electrophoresis step to differentiate between intact DNA and single or double strand DNA damage. Both assays are rapid and sensitive, and allow the evaluation of DNA fragmentation on a few sperm. The disadvantages are the lack of standardized protocols and the need for software to conduct image analysis.

The In Situ Nick Translation (ISNT) is a modified form of TUNEL, which utilizes incorporation of biotinylated-dUTP at the ssDNA in a reaction catalyzed by template dependent enzyme, DNA polymerase 1 (DNA Pol 1). ISNT can only be used for single strand breaks and has a very dynamic range and lacks sensitivity compared with other assays.

The DNA Breakage Detection-Fluorescence in Situ Hybridization (DBD-FISH) test permits any sites of DNA damage in the sample genome to be analyzed *in situ* using an alkali DNA unwinding solution. Cells are stabilized in agarose beads, and the incubation with the unwinding buffer leads to the presence of single-stranded DNA in the sample that can be hybridized with the appropriate probes. The technique has been successfully used to test the DNA fragmentation levels in sperm samples but it is still in development and no field results have been shown yet.

Finally, the Single-cell pulsed-field gel electrophoresis technique is an assay that is still being developed and that allows detection of the early stages of DNA fragmentation determining quantitatively the number and size of DNA fragments derived from a single sperm nucleus.

## 3. Types of DNA Damage and Base Modification

**Figure 1:**
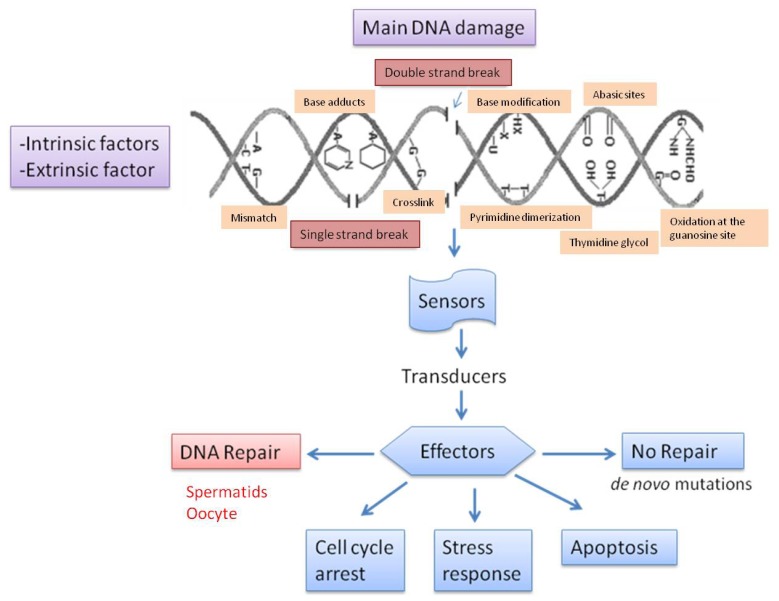
Summary of the major causes of DNA damage due to intrinsic and extrinsic factors Reproduced and modified with permission from Menezo *et al*. [38].

In mammalian germ cells we can encounter several types of DNA damage. Most damage originates in the male gamete. A summary of the major causes of DNA damage is shown in Figure 1. DNA fragmentation is characterized by both single (SSB) and double DNA strand breaks (DSBs), and it is particularly frequent in the ejaculates of subfertile males. TUNEL and Comet assays are able to detect single and double strand DNA breaks [33]. Sperm DNA fragmentation induced by oxidative attacks like the hydroxyl radical and ionizing radiation results in the formation of 8-OH-guanine and 8-OH-20-deoxyguanosine (8-OHdG) at a first stage and single-stranded DNA fragmentation thereafter. Hydroxyl radical formation may result in the indirect induction of double-stranded sperm DNA damage through the activation of sperm caspases and endonucleases [34].

DNA double-strand breaks are extremely harmful lesions that can lead to genomic instability and cell death if not properly repaired. There are several options for a cell that is facing DNA damage (Figure 1). However, although DNA damage may be repaired, fertilization of an oocyte by a spermatozoon with extensive double-stranded DNA fragmentation can be virtually not repairable and incompatible with normal embryo and fetal development [35]. Genome integrity controlled by means of a sophisticated cellular network, the DNA damage response (DDR), where a series of proteins are mobilized in response to genotoxic stress. Three distinct protein complexes act as sensors, transducers and effectors of DDR induced by DSBs [36]. Many components of these three layers interact with each other and converge toward different outcomes depending on the severity of the damage and on the cell type. The activation of checkpoints slows down cell cycle progression until lesions are resolved. If unrepaired DSBs persist, cells can undergo apoptosis or senescence to prevent the accumulation of potentially tumorigenic mutations. If all the damage responses fail, *de novo* mutations will appear [37].

## 4. Causes of DNA Fragmentation

### 4.1. Intrinsic Causes

Abnormal sperm chromatin/DNA structure is thought to arise from the following potential sources.

#### 4.1.1. Deficiencies in Recombination during Spermatogenesis

Errors during recombination usually lead to cell abortion. Meiotic crossing-over is associated with the genetically programmed introduction of DNA breaks by specific nucleases. Opportunities for DNA–DNA or DNA–protein cross-linking are greater in the highly compacted chromatin of mature sperm than in somatic cells. In recent studies catechol estrogens have been shown to form dimers that covalently cross-link the DNA so that it becomes completely resistant to the decondensation protocols employed in a comet assay including treatment with reducing agents, detergents and broad spectrum proteases [39]. Significantly, severely cross-linked chromatin is commonly encountered in populations of defective spermatozoa [40] although the molecular basis of such super-stabilization is still unknown.

#### 4.1.2. Abnormal Spermatid Maturation

DNA breaks are necessary for transient relief of torsional stress, favoring casting off of the nucleosome histone cores, and aiding their replacement with protamines. Chromatin packaging includes a step that needs endogenous nuclease activity to loosen the chromatin by histone hyper-acetylation and introduction of breaks by topoisomerase II, capable of both creating and ligating breaks. Chromatin packaging around the new protamine cores should be completed and DNA integrity restored during epididymal transit [41]. However, if the temporary breaks are not repaired, DNA fragmentation in ejaculated spermatozoa can occur.

#### 4.1.3. Protamine 1 and 2 Ratios

Variations in sperm protamine expression are associated with male infertility. In humans, during late spermiogenesis, 85%–95% of histones are replaced sperm through a multi-step process [42]. First, the histones undergo hyperacetylation; then they are replaced by testes-specific variants of the histones, followed by their replacement with transition proteins. The transition proteins are then replaced by protamines 1 and 2 (P1, P2). P1 and P2 are normally expressed in a 1:1 ratio in human sperm, and provide a tight packaging of the sperm DNA, resulting in a compaction of the nucleus and cessation of gene expression [43]. Abnormally high and low P1/P2 ratios are proven to be associated with increased sperm DNA fragmentation, lower fertilization rates, poor embryo quality and reduced pregnancy rates [44,45].

#### 4.1.4. Abortive Apoptosis

An alternative etiology for the DNA DSBs in the spermatozoa of infertile males can arise through the abortive apoptotic pathway. As male germ cells transform into highly differentiated spermatozoa, they progressively lose their capacity to undergo programmed cell death in the form of apoptosis since these cells are transcriptionally and translationally silent. Instead of engaging in a complete apoptotic response leading to cell death, differentiating haploid germ cells are thought to undergo a restricted form of this process leading to DNA fragmentation in the nucleus whereas retaining the capacity to differentiate into mature functional spermatozoa that may still be capable of fertilization [46].

#### 4.1.5. Oxidative Stress

Reactive oxygen species (ROS) play an important and positive role by modulating cell proliferation, differentiation and function, but also may have negative effects because these species are highly reactive and may damage any cell structure, including the DNA molecule [47,48] In the semen of fertile males the amount of ROS generation is properly controlled by seminal antioxidants. The pathogenic effects of ROS occur when they are produced in excess of the antioxidant capabilities of the male reproductive tract or seminal plasma. Damaged sperm chromatin is known to contain base adducts. The major DNA adducts found in human sperm DNA are 8OHdG and two ethenonucleosides (1,N6-ethenoadenosine and 1,N6-ethenoguanosine). SSB is a direct consequence of oxidative attacks on sperm DNA, while the DSBs probably arise from exposure to 4-hydroxy-2-nonenal, a major product of lipid peroxidation [34]. These findings, taken in conjunction with data revealing a high correlation between DNA damage and 8OHdG expression [49] suggest that the former is commonly the product of oxidative stress originating as a consequence of the apoptotic mechanisms described above, infiltrating leukocytes or failed antioxidant defense systems.

### 4.2. Extrinsic Causes

#### 4.2.1. Lapse of Time from the Ejaculation

Sperm DNA fragmentation is not a static parameter, since the longevity of sperm DNA decreases progressively with time following ejaculation. Several studies have reported that significant differences in the dynamics of DNA fragmentation exist among species and individuals. The reasons for such variation are unclear but they could be related to the protamine sequence. Results from 11 species cryopreserved, thawed, diluted in an appropriate extender for each species, and then incubated at 37 °C showed that the expression of protamine 2 significantly enhanced the likelihood of sperm DNA fragmentation. Also, greater numbers of cysteine residues in protamine 1 tended to confer increased sperm DNA stability [50].

#### 4.2.2. Collection Methods, Extenders and Post-Ejaculation Treatments

Semen collection methods (artificial vagina compared to electroejaculation), season in which the semen is collected (breeding season compared to non-breeding season), extenders and pre-treatment procedures (washing protocols) have an effect on sperm DNA quality. Also, differences are found between species and among individuals of the same species [51]. It has been proven that ejaculates collected by artificial vagina have a lower percentage of sperm DNA fragmentation. Also, sperm samples collected during the breeding season in specific extenders presented better sperm quality [52].

#### 4.2.3. Sperm Preparation Techniques for ART

It has been published that techniques to help fertilization *in vitro* like sperm capacitation and acrosome reaction do not damage sperm DNA nor affect histone content. According to the literature, both techniques support a physiological remodeling of non-damaged human sperm chromatin and modifications are probably interlinked and help prepare chromatin for post-fertilization events [53]. Moreover, a combination of density gradient and swim-up techniques has been recommended for IVF as sperm prepared by this method have been found to have higher rates of motility and reduced DNA fragmentation [54,55].

#### 4.2.4. Storage Temperature and Cryopreservation

The effect of storage temperature and cryopreservation on sperm DNA has been studied in various species. It is known that temperatures between 5 °C and 15 °C maintain the DNA intact for longer periods of time than higher temperatures like 20 or 37 °C, and that the higher the temperature the more drastic will be the rise on SDF on stallions, rabbits, dogs and bulls [56,57]. However, on species like elephants, cooled semen markedly reduces fertility and sperm DNA stability during incubation at 37 °C [58]. On the other hand, although cryopreservation of sperm is useful to preserve male fertility before therapy for malignant diseases, vasectomy or surgical infertility treatments in humans, and to use in artificial reproduction programs for the genetic improvement in animal species, this technique may lead to deleterious changes of sperm structure and function. Different studies on sperm DNA fragmentation dynamics before and after sperm storage show that cryopreservation is an important issue that must be considered since it can decrease the DNA longevity [59,60]. The differential and species-specific dynamic loss of sperm DNA quality as observed in all the mammalian species so far analyzed may have a negative incidence in the reproductive outcome [50].

#### 4.2.5. Mechanical Conditions—Sex-Sorting

New techniques for sperm selection are being implemented in the sperm market. One of them is the sex selection of sperm to determine the gender of the offspring with over 90% accuracy. Studies have shown that sex sorting does not affect the quality of DNA on species like deer [61] and boar [62]. However, its effect is still to be determined on bovine sperm since studies have shown that, although DNA fragmentation right after thawing is higher in conventional than in sex-sorted sperm samples, a reduced DNA longevity in sex-sorted spermatozoa was detected when the samples were incubated for 48 h [63].

#### 4.2.6. Post-Testicular Oxidative Stress

Recent studies show that immature sperm, which produce high levels of ROS, can induce DNA damage in mature sperm. This damage would be produced after spermiation from the seminiferous tubules to the epididymis and after ejaculation [64]. The ROS can damage sperm DNA directly or indirectly through the activation of sperm caspases and endonucleases. This is consistent with the fact that co-centrifugation of immature sperm (that produce high levels of ROS) with mature sperm results in the induction of sperm DNA fragmentation in mature sperm, because under these conditions, mature and immature sperm are in close contact. And also consistent with the fact that the *in vitro* exposure of mature sperm to ROS results in significant DNA damage [65]. The epithelial cells from the epididymis could also play an active role in ROS-induced DNA damage, through ROS such as the hydroxyl radical or nitric oxide [66] or through the activation of sperm caspases and endonucleases by factors such as high temperature [67]. In most cases, this damage could theoretically be prevented with the use of antioxidants. This is supported by the study by Greco [32], in which the use of antioxidants resulted in a significant reduction in the levels of sperm DNA fragmentation. However, some antioxidants, like vitamin C, may increase chromatin compaction by reducing disulfide cross-linking in sperm protamines [68].

#### 4.2.7. Varicocele

Varicocele occurs in approximately 15% to 20% of the general human male population and it is the most common cause of poor semen production and decreased semen quality. It has been demonstrated that human patients with varicocele have a significantly higher DNA fragmentation index. Studies show that varicocele samples contain a higher proportion of spermatozoa with abnormal DNA and immature chromatin than those from fertile men as well as infertile men without varicocele. Therefore, varicocele results in the production of spermatozoa with less condensed chromatin and this is one of the possible causes of infertility due to varicocele [69,70].

#### 4.2.8. Bacterial Infections

Human patients with genitourinary infection by *Chlamydia trachomatis* and *Mycoplasma* have increased sperm DNA fragmentation in comparison with fertile controls. This increase is proportionally greater than the influence on classical semen parameters and could result in a decreased fertility potential. Antibiotic therapy appears to be important in providing a remedy for infection-induced high DNA fragmentation levels [71]. This effect has also been seen in other animal species; results show a significant increase in DNA damage due to bacterial growth after ejaculation [72].

#### 4.2.9. Age

Some studies in humans suggest that neither the fertilizing capacity and routinely assessed semen parameters nor the amount of spermatozoa with fragmented DNA are affected by male age [73,74]. However, other studies demonstrate a significant increase in sperm DNA damage with age, and suggest that DNA Fragmentation is significantly lower in men under 35 years [75,76].

#### 4.2.10. Abstinence

One of the first studies to report on the effect of abstinence on sperm quality after specified target days of abstinence in humans showed that sperm numbers and semen volume increased with duration of abstinence. Abstinence did not influence pH, viability, morphology, motility, or sperm DNA fragmentation. However, a short (24-h) abstinence period negatively influenced chromatin quality [77]. On the other hand, later studies suggest that lower baseline levels of SDF are observed after shorter periods of abstinence between ejaculations (24 h and 3 h) than those recommended [78].

#### 4.2.11. Temperature of Testis

Testicular function is temperature dependent, and, in some animal species, the position of the testes in the scrotum ensures that they are kept at between 2 and 8 °C below core body temperature. A recent publication, using a mouse model, reported that DNA strand breaks were present in spermatocytes recovered from testes subjected to 40 or 42 °C. Also, paternal heat stress resulted in reduced pregnancy rate, placental weight and litter size [79].

#### 4.2.12. Reaction to Clinical Procedures, Medicines or Vaccines

It has been previously reported that exposure to chemotherapy and radiotherapy may also result in the induction of sperm DNA fragmentation. It is generally believed that cancer treatments adversely affect male fertility and that reduction of sperm output arises from the cytotoxic effects of chemotherapy or radiotherapy on the spermatogenic epithelium [80]. Medical treatments like paroxetine [81] induced abnormal sperm DNA fragmentation in a significant proportion of human patients. Finally, some vaccines affect negatively sperm DNA quality, it has been proven than Miloxan increases the percentage of sperm cells with fragmented DNA by 10-fold on ram. However, this negative impact appears to reversible and therefore collection of sperm samples should be avoided until at least one month after vaccination [82].

#### 4.2.13. Exposure to Environmental Chemicals

The toxicology literature indicates that certain environmental contaminants can, at least at experimental doses, induce sperm DNA fragmentation [83,84] and/or induce oxidative stress [85]. At least 200 exogenous chemicals can be measured in most people at any given time, but very few chemicals have been evaluated specifically for their sperm DNA damaging potential. Previous studies have provided evidence of an association between exposure to high levels of air pollution and increased DNA damage in human sperm [86].

## 5. Repair of Sperm DNA Damage

Mammalian germ cells encounter several types of DNA damage. Most damage originates in the male gamete. A summary of the major causes of DNA damage is shown in Figure 1.

There are three options for a cell that is facing DNA damage. The first is to activate the apoptotic pathway; this activation leads to cell death [87] that will destroy cells and impair viability. The second option is to tolerate the lesion; this option may lead to mutations in the next generation. The third and best option is to repair the lesion. A network of DNA damage response (DDR) mechanisms protects organisms and almost completely repairs damage in a short period of time to provide the maintenance of genomic integrity. The main repair mechanisms operating in the mammalian germline cells are: nucleotide excision repair (NER), base excision repair (BER), mismatch repair (MMR), post replication repair (PRR) and DNA double strand break repair (DSBR). During the last years, significant new insights have been gained into the mechanism and biological relevance of DSBs repair in relation to genome stability. DSBs are a highly toxic DNA lesion, because they can lead to chromosome fragmentation, chromosome domain loss, translocations or other genome rearrangements [88]. Currently, there are relatively few publications that summarize the basic information and new findings on DNA repair mechanisms used in mammalian germ cells. Most of them are centered in other cell types and related to carcinogenesis. In the present article, we review the studies that discuss the main DSBR operating in the female and male germ cells.

Along evolution, several pathways have evolved for the repair of these DSBs. The most important DSB repair mechanisms in mammalian cells are non-homologous end-joining and homologous recombination. By using a non-damaged repair template, homologous recombination ensures accurate DSB repair, whereas the untemplated non-homologous end-joining pathway does not. Although both pathways are active in mammals, the relative contribution of the two repair pathways to genome stability differs in the different cell types. Given the potential differences in repair fidelity, it is of interest to determine the relative contribution of homologous recombination and non-homologous end-joining to DSB repair. In this section, we focus on the biological relevance of DSB repair in mammalian germ cells. Hence, we are going to divide the repair strategies in repair mechanisms carried out by spermatid cells (round and elongated spermatids) and in fertilized oocytes.

### 5.1. Repair during Spermiogenesis

**Figure 2:**
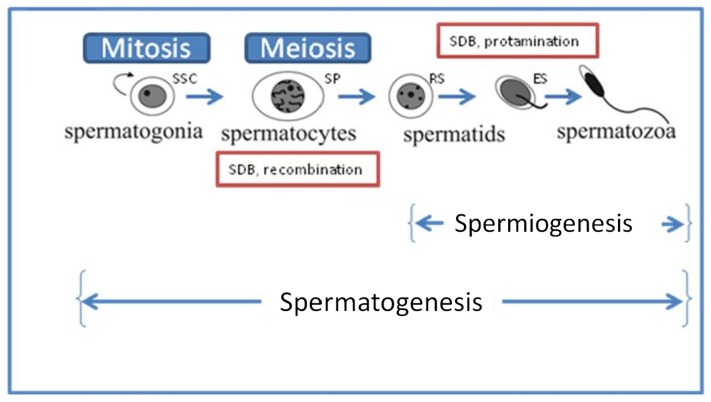
Diagram of spermatogenesis initiated with the mitotic proliferation of spermatogonia (SSC; 2n) followed by first meiotic (I) division resulting in the formation of primary and secondary spermatocytes (SP). Through meiotic division (II), 2° SPs generate haploid round spermatids (RS) entering the differentiation process of spermiogenesis to produce elongating spermatids (ES) and mature sperm.

Spermatogenesis consists of three distinct phases (Figure 2). First, the spermatogonia (SSC) go through a series of mitotic amplifying divisions and differentiate into primary spermatocytes (SPs). In the second phase, spermatocytes undergo meiotic recombination giving rise to haploid spermatids. The third phase, termed spermiogenesis, involves the rearrangement of the cytoskeletal structure, transforming round spermatids (RS) into mature spermatozoa. Hence, the maturation of germ cells involves a remarkable genomic reorganization that requires an extraordinary process of chromatin remodeling to generate haploid gametes [89]. During this process, histones are removed from the DNA and are first replaced by transition proteins TP1 and TP2, and then by protamines P1 and P2. Only a small, but apparently well-defined fraction of the sperm genome remains histone-associated in mature sperm. Abnormally increased amounts of histones in sperm are associated with decreased fertility and increased risk of embryonic failure after fertilization [90]. Therefore, histone retention and protamine deficiency in sperm are hallmarks of certain forms of idiopathic infertility [91–93], but the genetic and mechanistic causes underlying this defect remain enigmatic.

The transition from histone- to protamine-based chromatin during spermiogenesis is associated with a transient occurrence of physiological DNA strand breaks [94,95]. These transient DNA strand breaks appear in the whole population of elongating spermatids during mid-spermiogenesis [96], which likely permit topological changes associated with DNA relaxation during nucleoprotein exchange in spermiogenesis, have been attributed to the activity of topoisomerase II beta (TOP2B) [97]. Most likely, these transient breaks are required to support the change in DNA topology associated with chromatin remodeling at these steps. Histones hyperacetylation is also coincident with the DNA strand breakage steps and may represent a necessary condition for strand breakages and permit the removal of DNA supercoils. During the chromatin remodeling in spermatids, the combined DNA-condensing activities provided by the basic transition proteins and protamines may optimize the strand repair process emphasizing the link between altered sperm DNA condensation and DNA fragmentation. The mutagenic potential of these events may have been overlooked as it may result in fertility and/or developmental problems.

DNA double-strand breaks are extremely harmful lesions that can lead to genomic instability and cell death if not properly repaired. In contrast to the dynamic field of DNA repair in nuclei of somatic cells [98], insight into DNA repair mechanisms in the mammalian germ line is only slowly developing [99].

**Figure 3:**
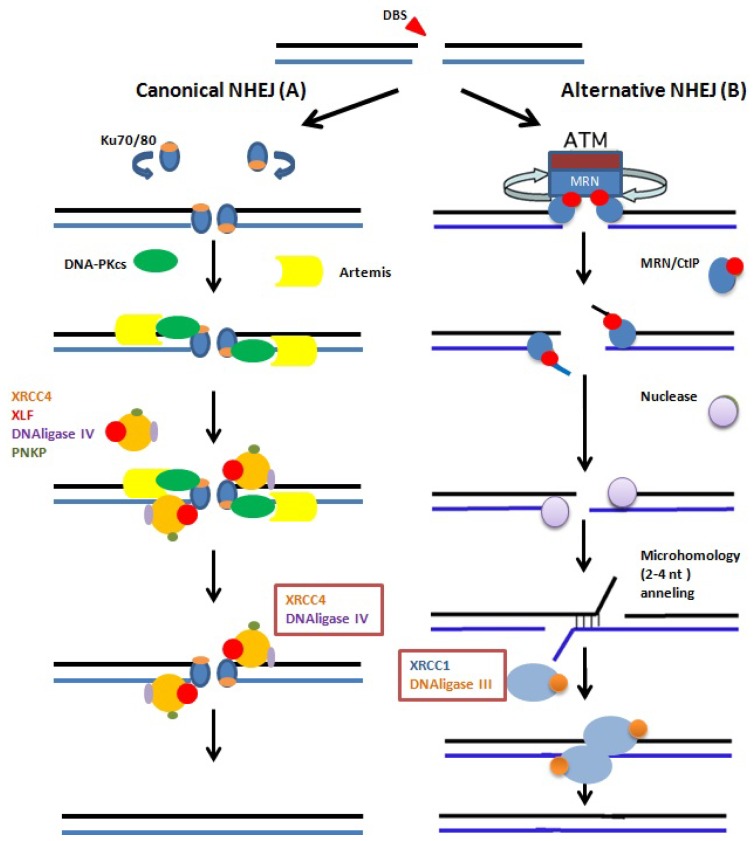
The canonical and NHEJ-alternative pathways. (**A**) The canonical NHEJ pathway, involving KU and XRCC4, can seal double-strand ends, even distal and non-fully complementary ends, in a conservative fashion; (**B**) In the alternative pathway, the main event is extended deletion at the junction, generally associated with the use of internal microhomologies distant from the ends. And XRCC1 and DNA ligase-III are used for strand ends ligation.

There are at least three pathways that are responsible for repairing DNA DSBs in mammalian cells: non-homologous end joining, homologous recombination and alternative non-homologous end joining [100]. In higher eukaryotes, DSBs are predominantly repaired by the NHEJ pathway involving the DNA-PKcs which is recruited by the Ku70, and Ku80 proteins to the site of damage and subsequently, both end-positioned Ku and DNA PKcs mediate the recruitment of XRCC4/DNA ligase IV complex (Figure 3A) which is responsible for the ligation step [101]. In contrast, spermatogenic cells differ considerably in their DSB repair kinetics, and the lack or deficiency of certain repair proteins has clearly different effects on the DSB repair capacities of distinct germ cells [102]. Several lines of evidence suggest that alternative and less well defined back-up pathways may contribute to physiological and pathological DSB repair [103,104]. This back-up pathway requires the synaptic activity of PARP-1 and the ligation activity of the XRCC1-DNA ligase III complex (Figure 3B), proteins involved in base-excision and single-strand repair [105]. Recent work by Ahmed *et al*. suggests that round spermatids utilize this alternative back-up pathway to repair radiation-induced DSBs [106]. Rube *et al*., in 2011 [102] confirm that DNA-PKcs deficient round spermatids of SCID mice reveal nearly identical DSB repair kinetics to those of DNA-PKcs proficient spermatids, indicating that the slow and incomplete DSB repair in round spermatids is independent of the classical DNA-PK dependent NHEJ. Furthermore, in DNA-PKcs-deficient SCID mice, DSB repair capacities in SSCs were clearly more efficient than in somatic tissue cells, suggesting that the DNA-PK dependent NHEJ pathway does not play a major role in DSB repair in SSCs. A potential explanation for this is that the various spermatogenic cell types are characterized by clearly different chromatin compositions, and therefore may require different repair proteins and repair mechanisms to restore the integrity of their genome.

There is increasing evidence that chromatin structure strongly influences DNA repair processes [107–109]. Spermatogenesis is characterized by spectacular chromatin remodeling processes in which somatic histones are sequentially replaced by testis-specific variants during pre-meiotic, meiotic, and post-meiotic stages of germ cell differentiation. The spermiogenesis following the meiotic division is characterized by the histoneto-protamine exchange, resulting in condensed DNA packaging of the paternal genome. These extensive alterations in chromatin architecture certainly have a profound impact on DNA repair mechanisms, as modulation of the chromatin structure itself is an important precondition for the recruitment and function of DNA repair proteins. Phosphorylation of H2AX was the first chromatin associated event shown to occur at DSBs and is believed to function as a platform for the recruitment and/or retention of DNA repair and signaling molecules at sites of DNA damage. At least one of these components, MDC1, binds directly to the phosphorylated C-terminal tail of histone H2AX [110]. But in spermatogonias (SSCs), which are characterized by a complete lack of compacted heterochromatin, DNA damage detection and signaling is mediated in the absence of the transducer complex H2AX/MDC1, and radiation-induced DSBs are repaired predominantly by DNA-PKcs-independent mechanisms. Based on these findings, they have suggested that alternative forms of end-joining operate in SSCs to restore their genomic integrity. In response to genotoxic insults, effective cell cycle checkpoints in the differentiating progeny, but not in SSCs themselves, eliminate damaged cells by apoptosis, thereby ensuring that only intact genetic information is transmitted to subsequent generations.

Male germ cells are highly distinct from somatic cells in their chromatin organization. Rube *et al*., in 2011 [102] showed that cell type-specific chromatin compositions are associated with the recruitment and function of certain DNA repair components. The orderly sequence of nuclear protein binding to DNA may facilitate the repair of these transient DNA nicks. In agreement with such a role for these basic proteins *in vivo*, mice with haploinsufficiency for the protamine 2 were shown to have a much higher frequency of sperm with damaged DNA as determined by the Comet assay [111]. In addition, mice harboring a double deletion of both the Tnp1and Tnp2 genes displayed a clear persistence in DNA strand breaks in those spermatids where the condensation process has been clearly altered by the deletions [112]. It is therefore not surprising that, in many instances, an alteration in the condensation state of the sperm head has been correlated with the presence of DNA strand breaks [113–116]. Aside from an alteration in the condensation process, DNA breaks found in mature sperm may result from failure in the postmeiotic DNA repair or from an increase in reactive oxygen species. An alteration in DNA condensation may also offer an opportunity for endonucleases to attack the DNA phosphate backbone due to the lack of proper protection by the nuclear basic proteins [117].

To complete their development, the spermatids have to pass through the epididymis, where the process of disulfide cross-linking takes place, and the induction of sperm DNA fragmentation in the epididymis could be related to their genomic quality. Most likely, the sperm that have higher levels of DNA damage would be those that acquire lower levels of disulfide cross-linking in their chromatin during the process of sperm maturation in the epididymis. That is, in addition to the screening mechanism exerted by the Sertoli cell during the process of spermatogenesis, there would be another screening mechanism at the level of the epididymis directed to eliminate genomically defective sperm [118].

A better characterization of the enzyme activity involved at theses repair mechanisms is necessary as this may represent a very sensitive process where alterations in the genetic integrity of the male gamete may arise and persist up to the mature spermatozoa.

### 5.2. Repair in the Fertilized Oocyte and during Early Embryonic Development

Sperm with DNA fragmentation still has fertilizability and developmental potential. Depending on the level of sperm DNA fragmentation, three situations can be expected: In some cases, the oocyte repair machinery is not sufficient to repair DNA damage, and the embryo may fail to develop or implant in the uterus or may be aborted naturally at a later stage (uncompensable damage). In other cases, the oocyte repairs the DNA strand breaks before the initiation of the first cleavage division, and this sperm is then able to generate normal offspring (compensable damage). In the worst and last scenario, deletions or sequence errors may be introduced because of partial oocyte repair, and abnormal offspring may then result (partial compensable damage). It has been reported that 80% of the novo structural chromosome aberrations in human are of paternal origin [119].

If DNA damage escapes gametogenic DNA repair or if damage occurs in the spermatozoa indeed to extrinsic factors, the damage can be successfully repaired during fertilization [120,121], which involves formation of pronuclei, DNA replication and pronuclear fusion leading to the formation of a zygote. DNA repair in the newly fertilized embryo is believed to rely entirely on the maternal mRNAs and proteins deposited and stored in the oocyte before ovulation. DNA repair genes have been shown to be expressed in the early stages of mammalian development. If the oocyte is not adequately equipped, or if the zygotic gene expression does not start at the correct time, the embryo will die [122]. Subsequent to this, DNA repair is expected to have a major impact on embryo development. It has been shown that human oocytes express DNA repair genes at high levels allowing low tolerance for DNA decays [123].

The cell cycle is much shorter in embryonic cells compared to adult cells [124]. The integrity of the genome is thus at greater risk during embryonic development and the efficiency of DNA repair at those early stages is of great significance for a given organism. In this section we will focus on the ability of the fertilized oocyte, zygote and embryo to repair sperm DNA damage.

#### 5.2.1. Sperm DNA Fragmentation Repair in the Fertilized Oocyte

The DNA repair transcripts that have accumulated in oocyte play a role during fertilization in controlling changes in chromatin remodeling and maintaining chromatin integrity. Experiments on rat and mouse zygotes have indicated recognition of DNA lesions and repair in the paternal chromatin after fertilization [125,126]. It is now clear that DNA damaged spermatozoa are able to reach the fertilization site *in vivo*[127], fertilize oocytes and generate early embryos both *in vivo* and *in vitro*.

During the first cell cycle of a fertilized egg, many changes occur at the genome level. Female meiosis is completed and the maternally inherited chromosomes are decondensed. The paternally inherited genome also decondenses and extensive chromatin remodeling occurs. Programming of both parental chromosome sets must then take place to create the embryonic genome and initiate embryo development [128].

Damage repair may occur after fertilization, but its mechanisms remain unknown. The biological impact of an abnormal sperm chromatin structure depends on the combined effects of the extent of DNA or chromatin damage in the spermatozoa and the capacity of the oocyte to repair that damage [129]. Ahmadi *et al.*[130] suggests that the oocyte has the capacity to repair DNA damage of sperm when it is damaged by less than 8%. The ability of the oocyte to repair DNA damage in the fertilizing spermatozoon is also going to depend on the type of sperm DNA damage. As indicated previously, sperm DNA fragmentation can be classified as single-stranded and double-stranded. In general, single-stranded DNA damage is easier to repair than double-stranded DNA damage, although there is evidence that polymerases can also repair double-stranded DNA damage.

Jaroudi *et al*., in 2009 [131], with microarray analysis detected large numbers of repair genes indicating that all DNA repair pathways are potentially functional in human oocytes and blastocysts. The higher mRNA level for most repair genes in oocytes compared with blastocysts ensures sufficient availability of template until embryonic genome activation. Overall, mRNA templates coding for 12/19 genes involved in HR repair and 3/6 genes involved in NHEJ were detected in both the MII oocyte and blastocyst groups. Thus DSBR via the HR pathway seems to be active in MII oocytes and blastocysts. This is expected for the oocytes (as HR is active during M phase) and for the blastocysts since HR has greater fidelity of DNA repair than NHEJ and thus may be the preferred mechanism for DSBR. NHEJ does not require sequence homology between the DNA ends as a prerequisite for ligation. As a result, this process can be error-prone. In contrast, HR is generally considered to be an error-free process. Despite the fundamental differences between pathways of DSB repair, it has become clear that these pathways compete to repair DSBs. However, how a cell chooses whether to repair a DSB through NHEJ or HR, is still relatively unknown and has become an active area of investigation

There is little data as to whether it is possible to increase DNA repair capacity in oocytes. Some effectors of oocyte competence may be active. One fact is clear, with increasing maternal age the mRNA stored in oocytes decreases as well as the efficiency of DNA repair [132].

#### 5.2.2. Sperm DNA Fragmentation Repair in the Zygote

DNA repair in the zygote is a maternal trait. The maternal to zygote transition in gene expression in the mouse occurs at the 2-cell stage [133]. Hence, the repair genotype of the mother is decisive for the repair capacity of the zygote. This point is elegantly illustrated by the use of maternal DNA repair mutants in the repair process in the zygote [125,134,135]: The maternal transcripts and proteins to support the zygote’s development until embryonic genome activation (EGA).

Paternal DNA damage may be translated into chromosome aberrations at the first metaphase in the zygote [136]. DSBs are probably the most important type of DNA damage as they induce chromosomal instability. DSBs repair is performed during zygotic cell cycle by at least two sub-pathways: Non-homologous end joining (NHEJ) and homologous recombination (HR). Differentiated somatic cells often resolve DSBs by NHEJ, whereas embryonic stem cells preferably use HR [125]. These pathways have different relative importance. HR is active during the late part (S/G2) of the cycle, when sister chromatids are available as a template for repair [137], whereas NHEJ, predominantly active in G1. Thus, the phase of the cell cycle determines by and large what type of repair mechanism is operative. HR seems to be the most active system in mammalian cells, when replication fails or blocks (replication fork or replication stalling) [138]. The precise replication of the genome during S-phase is of fundamental importance especially in the zygote. Also a role for poly(ADP-ribose) polymerase (PARP) in dsDNA break repair in the zygote was strongly suggested by Matsuda and co-workers [136].

#### 5.2.3. Sperm DNA Fragmentation Repair in during/after Implantation Embryo

The presence of unrepaired DNA damage above a critical threshold in embryos generated *in vivo* and *in vitro* has been postulated to explain the failure in embryo development observed after embryo implantation in embryos with a normal karyotype. Recent studies suggest that this type of damage is expressed during/after implantation and has been characterized as late paternal effect [139,140]. There are also indicators of high levels of DNA damage in a sperm sample with failure to obtain blastocysts [141] and it is believed that some loss of preimplantation embryos occurs between postembryonic genome activation and the blastocyst stage [142,143]. The short G1 and G2 phases in rapidly dividing blastomeres support the assumption that HR is the dominant DSBR mechanism in the blastocyst. The first step in HR involves RAD52, which competes with KU to direct DSBR toward HR rather than NHEJ [144,145]. Many animals lacking DNA repair enzymes are not viable with preimplantation death being a common result; however, much of the embryonic loss takes place around implantation, showing the necessity for DNA repair ability when embryonic cells proliferate rapidly and differentiate [146]. Many DNA repair genes involved in DNA damage response pathways seem to be expressed in post-implantation mammalian embryos, especially from the mid-gestational stage onwards. At this stage spatial patterns of expression in the embryo become apparent for some DNA repair genes [147].

The capacity of the mammalian embryo to respond and repair damaged DNA and its selective sensitivity to specific lesions is still not well understood. Many gaps exist in our current knowledge concerning the precise roles and expression timings of several DNA repair genes in the early stages of embryonic development. The observed developmental stage-specific variations in DNA repair gene expression transcripts and proteins point out the complexity of the regulation of these pathways during development

Summarizing, even if the fertilizing spermatozoon carries DNA damage in its genome, the oocyte, zygote and blastocyst could repair this damage and, therefore, it would be of no consequence for embryo and fetal development. However, we cannot determine whether the early embryonic development stages would be capable of repairing this damage. In addition, DNA fragmentation tests currently available cannot provide information concerning the “reparability” of sperm DNA damage. Hence test, which will predict the reparability of sperm DNA fragmentation, should be developed. In our laboratory, the 2D-COMET has been introduced as a novel test that can help evaluate the impact of double-stranded sperm DNA damage on ART pregnancy outcome and improve the specificity of DNA fragmentation testing [33].

## 6. Conclusions and Future Directions

Infertility is a significant issue, with approximately 20% of men in western countries affected [148]. As meiotic recombination, the protamination involves DSB generation; it seems likely that certain DNA damage response (DDR) defects would cause infertility. Indeed, DDR signaling is readily detectable during human spermatogenesis [149], and various inherited DDR deficiencies are characterized by infertility or sub-fertility. A significant proportion of human infertility might therefore be caused by DDR deficiencies.

In the past two decades, the understanding of mechanisms of DNA damage repair has greatly advanced due to the extensive studies of the unicellular eukaryote *Saccharomyces cerevisiae*, as well as various animal models and cancer-susceptibility in humans but little is known about repair in germ line cells. Accumulating experimental results have begun to decipher the function of various checkpoint sensors, mediators and effectors as well as their relationship with protein factors participating in different DNA repair mechanisms.

Further study of these pathways will enhance our understanding of repair sperm DNA damage and could provide a basis for the effective prevention and treatment of infertility.

Although the usefulness of SDF assessment using the available tests is accepted, insufficient resources have been available to develop standardized tests and protocols that could lead to universally accepted clinical thresholds. This shall be one of the goals to really understand the role of SDF in reproductive outcome. Associated with the lack of useful prognostic tests is the lack of improvement in assisted conception success rates despite thirty years of worldwide use. International collaborations should be initiated to develop agreed protocols and establish thresholds. The second issue of interest that shall need further investigation is the repair capacity of the oocyte of damaged spermatozoa. This is of crucial interest since unrepaired or mismatched DNA repaired may give rise to undesirable problems of embryo cleavage and development.
